# Stress hormone response to the DEX–CRH test and its relation to psychotherapy outcome in panic disorder patients with and without agoraphobia

**DOI:** 10.1038/s41398-017-0081-7

**Published:** 2018-02-02

**Authors:** Susann Wichmann, Stefan R. Bornstein, Thomas Lorenz, Katja Petrowski

**Affiliations:** 1 Department of Psychology and Psychotherapy, University Witten/Herdecke, Alfred-Herrhausen-Straße 50, 58448 Witten, Germany; 2 Department of Internal Medicine, University Hospital Carl Gustav Carus Dresden, Technische Universität Dresden, Fetscherstraße 74, 01307 Dresden, Germany; 3 Department of Psychotherapy and Psychosomatic Medicine, University Hospital Carl Gustav Carus Dresden, Technische Universität Dresden, Fetscherstraße 74, 01307 Dresden, Germany

## Abstract

This study tested whether the hormonal stress response to the DEX–CRH test may be predictive of the psychotherapy success for panic disorder (PD). Thirty-four patients diagnosed either with agoraphobia with PD or PD without agoraphobia were subjected to cognitive behavioural therapy (CBT). Patients (pre-therapy) and healthy volunteers were exposed to the DEX–CRH test. Blood samples were taken for cortisol and adrenocorticotropic hormone (ACTH) assessment. Established panic-specific questionnaires were handed out for the pre-therapy and post-therapy evaluation of disease severity (with reference to panic beliefs and agoraphobic cognitions, fear of bodily sensations, agoraphobic avoidance behaviour). Repeated measures ANCOVA were conducted for the analysis of the pre-therapy hormonal response, and Pearson’s correlation analysis to test for associations with the psychotherapy outcome. Data analyses revealed large effect sizes for CBT in the clinical measures (*η*^2^ ≥ 0.321), main effects of time for cortisol and ACTH with no differences between both groups, and significant associations between cortisol release and agoraphobic cognitions for the patients. PD diagnosis had no impact on the hormonal response. However, those patients with higher cortisol release showed less improvement after CBT (significantly for agoraphobic cognitions). Clinical implications of these findings are the prediction of the therapy success from a potential endocrine correlate whose persistency (if assessed repeatedly) during the treatment may predict (non-)response to the current treatment, possibly representing a decision support for a change in treatment to avoid the continuation of an inefficient treatment.

## Introduction

Panic disorder (PD) is an episodic mental disorder associated with substantial reductions in the quality of life^1^. PD is characterised by repeated acute panic attacks, concerns about future attacks, and their assumed implications to mental and somatic health followed by significant behavioural changes related to the panic attacks^2^. Patients with PD frequently consult emergency departments and their general practitioner for a variety of unspecific symptoms such as palpitations, dizziness, and shortness of breath. The fiscal costs of PD, and even subthreshold PD, were found to be the highest compared to other mental disorders^3^. Nevertheless, treatment for PD is available with proven efficacy; the economic returns for society far outreach individual benefits.

However, even though psychotherapy is the first choice treatment for PD^4^, there is still a proportion of patients who fail to demonstrate symptom remission following psychotherapeutic interventions^5,6^. To date, psychotherapy outcome research has put little emphasis on biological correlates of the therapy outcome but has focused primarily on symptom severity itself as negative predictor^7,8^, for comorbid mental disorders^9–11^ and motivation for psychotherapy^8^, both with no impact on the therapy success. Studies examining biological correlates within psychotherapy success prediction showed that PD patients with the lowest cortisol stress response showed the least improvement from psychotherapy (in response to individually feared situations^12^, and in response to the Trier Social Stress Test^13^). Until now, it has been uncertain if the cortisol stress response to the combined dexamethasone suppression/corticotropin-releasing hormone challenge (DEX–CRH) test, which is more cost-efficient than the Trier Social Stress Test (TSST), may be predictive of the psychotherapy outcome as well. However, the DEX–CRH test has more adverse effects than the TSST, for instance slight nausea, metallic taste, scraping feeling in the throat, sensation of heat and on rare occasions allergic reactions with shortage of breath^14^.

The combined DEX–CRH test is a widely used, reliable tool in psychiatric research to evaluate the neuroendocrine course and autonomic dysregulations of psychiatric disorders^15^. The DEX–CRH test requires the oral intake of 1.5 mg of a synthetic cortisol-like compound (dexamethasone) the night before investigation (11 pm) and the venous injection of 100 µg CRH at the day of investigation (3 pm). In mentally and somatically healthy individuals, the prolonged administration of dexamethasone inhibits the adrenocorticotropic hormone (ACTH) and cortisol release after additional administration of CRH. Therefore, the hormonal response to the DEX–CRH test provides an index for the normative functioning of the central glucocorticoid feedback regulation of the hypothalamic-pituitary-adrenal (HPA) axis (hence ACTH and cortisol suppression after prolonged DEX–CRH administration^)15^. Generally, changes in the activity of the HPA axis as the body’s major endocrine stress axis have been investigated extensively in reference to stress and anxiety or mood disorders (e.g., refs. 16,17,). Contradictory results exists for an altered HPA axis functionality with both evidence for a hypo- or hyper-reactive response pattern as a function of different mental disorders. Patients diagnosed with a major depression often showed an increased stimulation of the HPA axis (e.g., refs. 18–20), though a hypocortisolism pattern has also been reported, especially in elderly patients with a severe major depression diagnosis^17^. However, for anxiety, most evidence exists for a hypo-response pattern, for instance in patients diagnosed with PD (e.g., refs. 21–24) and post-traumatic stress disorder (e.g., refs. 25–27). Studies investigating the HPA axis responsivity with the DEX–CRH test in PD patients reported mixed findings. Most of the evidence was shown for a DEX–CRH non-suppression in PD patients who failed to show blunted ACTH and cortisol responses in comparison to healthy individuals^28–33^. In contrast to this non-suppression pattern, there is an evidence for lower cortisol levels in PD patients as seen in healthy individuals^22,34^. Besides, some studies failed to show substantial differences in the DEX–CRH hormonal stress response between the PD patients and healthy individuals, therefore providing evidence for a “normative” DEX–CRH suppression in PD patients (for ACTH^22^; for cortisol^35,36^; for both cortisol and ACTH^37,38^). However, the impact of the hormonal stress response to the DEX–CRH test on psychotherapy success in PD patients is relatively unclear. In depressed patients, DEX–CRH non-suppression has established itself as a reliable predictor for symptom relapse^39–44^. In a sample of PD patients investigated by Coryell et al. (1991), a DEX–CRH non-suppression before treatment was associated with more severe anxiety symptoms and disability after treatment with benzodiazepines^30^, yet were unable to find this association in an earlier sample^45^.

In the current investigation, patients in need of psychotherapeutic care for panic and agoraphobic symptomatology were exposed to hormonal stress induction using the combined DEX–CRH test at the beginning of the psychotherapeutic intervention. We aimed at investigating previous findings of an inverse relationship between the cortisol stress response upon the TSST, and the psychotherapy outcome could be replicated also for the DEX–CRH test which induces hormonal stress. Furthermore, we predicted that it would be possible to replicate an overall DEX–CRH non-suppression previously seen in patients diagnosed with PD. For this purpose, a control group including healthy volunteers was recruited to establish a possibility of comparison with the “normative” hormonal response to the DEX–CRH test.

## Methods

### Study participants

This study was part of a trial on psychotherapy success in patients diagnosed with PD with or without agoraphobia previously published^13^. The patient sample was recruited from May 2008 to May 2013 from the University Hospital of the Technische Universität Dresden, Germany, before the start of cognitive behavioural psychotherapy. Patients consulted the hospital on their own initiative or upon the recommendation of their general practitioner/psychiatrist. The healthy control participants were recruited via newspaper advertisements and matched to the patient sample by age and sex. General inclusion criteria were being aged 18 to 65 years and being fluent in the German language. Exclusion criteria included a history of substance abuse, benzodiazepine drug use, psychotic or bipolar disorder, post-traumatic stress disorder, eating or somatisation disorder, current pregnancy as well as any severe physical illness (e.g., cancer, a metabolic or autoimmune disorder) within the previous 2 years. The Structured Clinical Interview (SCID)^46,47^ for *DSM-IV-TR* diagnosis of mental disorders on axis I and II^2^ is routine standard diagnostic in the recruiting hospital and was conducted by trained clinical interviewers. Diagnoses were confirmed by an experienced psychotherapist (KP). Patients with a current primary diagnosis of PD without agoraphobia (F41.0) or agoraphobia with PD (F40.01), were included in the study. Secondary diagnoses of a mild major depressive disorder or specific phobia were permitted for inclusion in the patient group. Inclusion in the healthy control group was established using the DIA-X stem questions^48^ to confirm that the participants did not have a history of mental disorders.

Forty-three patients with PD and 34 healthy volunteers were screened with respect to the defined inclusion and exclusion criteria. Eight patients were excluded due to a current diagnosis of psychoactive substance use disorder (for details: *n* = 3 alcohol dependence syndrome, *n* = 2 comorbid alcohol and cannabinoid dependence syndrome, *n* = 1 cannabinoid dependence syndrome, *n* = 1 stimulants dependence syndrome, *n* = 1 multiple drug use) and one patient due to leukaemia, resulting in a sample of *n* = 34 patients with a primary diagnosis of either agoraphobia with PD or PD without agoraphobia (23 females, mean age ± SD: 35.50 ± 12.74) and *n* = 34 age-matched and sex-matched healthy volunteers (25 females, mean age ± SD: 33.82 ± 12.50). A total of *n* = 30 patients showed agoraphobia with PD and *n* = 4 patients showed PD without agoraphobia. Four patients were on antidepressant medication (selective serotonin reuptake inhibitor: Citalopram (*n* = 2), Paroxetine; tricyclic: Opipramol). Comorbid mental disorders were major depression (single episode: *n = *8; recurrent episode: *n = *4) and specific phobia (*n = *4).

All the study participants provided written informed consent. The study procedure was approved by the local Ethics Committee of the Medical Faculty of the Technische Universität Dresden, Germany (No# EK460230008).

### Cognitive behavioural therapy and clinical measures

Cognitive behavioural therapy (CBT) for the patients (not the healthy volunteers) was administered in individual and group sessions within 5 weeks of semi-residential care during which patients completed the following units: psycho-education referring to anxiety and in particular PD; explanation of the confrontation treatment rationale; exposure therapy including interoceptive exposure, therapist-accompanied and self-managed confrontation with feared situations, and, at last, cognitive therapy to modify anxiety-maintaining beliefs. The therapy was standardised based on the manual by Lang et al.^49^ Both the psycho-education and explanation of the treatment rationale were adapted as daily group sessions within the first 2 weeks. All the other units were administered as individual therapeutical contacts. All the study therapists were experienced in exposure therapy and cognitive behavioural techniques and were regularly supervised by an experienced psychotherapist (KP).

Information on sociodemographic variables, including sex, age, body mass index (BMI), and smoking status, as well as any somatic diseases and medication intake were assessed in a routine medical examination prior to the CBT. The participant’s physical activity was assessed non-standardised asking (a) “Are you regularly involved in sports (yes/no?), and (b) “How many hours per week have you been engaged in sports in the previous 3 months?”. The following self-report questionnaires were handed out in the German version both at the beginning of the study and after therapy. (1) The Panic & Agoraphobia Scale (PAS)^50^ was obtained to evaluate the global severity of the PD diagnosis. The PAS includes 13 items which belong to five subscales (panic attacks, agoraphobia, anticipatory anxiety, disability, and concerns about health) and must be answered on a 4-point Likert scale. A higher PAS total score indicates more severe panic symptoms. The PAS holds adequate test-retest-reliability (*r* = 0.78) and an internal consistency reliability of Cronbach’s *α* = 0.88^50^. With regard to criterion validity, the PAS demonstrates high correlations with the patient’s global impression of impairments in quality of life (*r* = 0.82), as well as with other panic-specific scales (*r* ≥ 0.68)^50,2^. (2)The Agoraphobic Cognitions Questionnaire (ACQ)^51^ is a self-report questionnaire assessing fearful panic beliefs and catastrophic cognitions about panic symptoms. 15 items must be answered on a 5-point scale anchored from (*“*thought never occurs”) to 4 (“thought always occurs”). The items are then averaged, both for the two subscales (loss of control, physical concerns) and the total score. (3) The Bodily Sensations Questionnaire (BSQ)^51^ measures fear of bodily sensations often occurring during panic attacks with 17 items to be answered on a 5-point Likert scale ranging from 0 (“not frightened”) to 4 (“extremely frightened”). The items are then averaged. (4) Agoraphobic avoidance behaviour was assessed using the Mobility Inventory (MI)^52^. The respondent indicates for 26 situations avoidance behaviour on a 5-point scale with response anchors ranging from 0 (“never”) to 4 (“always”) twice, once when confronted with the situation by him/herself and once when accompanied by another person. The items are then averaged separately for both. The German version of the three panic-specific questionnaires (2) to (4) meets high internal consistency with Cronbach’s α = 0.69 up to *α* = 0.87 as well as appropriate test-retest-reliability with *r* ≥ 0.74^53^. (5) The Beck Depression Inventory II (BDI)^54,55^ was handed out to evaluate the severity of depressiveness. This self-report rating inventory is composed of 21 groups of items matching the *DSM-IV-TR* major depression criteria^2^. Each item group consists of a list of four statements arranged in increasing intensity. The respondent is asked to choose the most appropriate alternative which best describes the way he or she felt during the previous 2 weeks. The inventory is internally consistent (Cronbach’s *α* ≥ 0.84) and concurrent to other self-report tools for depression (*r* = 0.72 to *r* = 0.89)^56^. Only the panic-specific questionnaires were handed out twice and the BDI was only handed out at the beginning of the study.

### DEX–CRH test and cortisol collection

The DEX–CRH test was performed according to the protocol published by Schreiber et al.^31^ and Heuser et al^15^. 1.5 mg Dexamethasone was administered orally at 11 pm. All the study participants came to the hospital at 2 pm the following day and rested in supine position with light reading permitted. They were instructed to refrain from eating, drinking, and smoking at least 2 h before as well as during testing. A venous catheter for blood sample collection was placed by a study nurse after an accommodation time of 30 min. Blood samples were taken 75 and 1 min prior to the intravenous injection of 100 µg CRH as well as immediately, 10, 20, 30, 45 and 60 min post-injection. Blood samples were stored at 4 °C. For the determination of plasma cortisol concentrations, blood was collected in serum gel monovette (Sarstedt, Nümbrecht, Germany) and immediately centrifuged at 4 °C and 3000 r.p.m. for 10 min. For the determination of plasma ACTH concentrations, blood was collected into tubes containing a mixture of trasylol and EDTA (Sarstedt, Nümbrecht, Germany). Then, plasma was stored at −80 °C and at −20 °C before being assayed for ACTH and cortisol. Plasma cortisol concentrations were determined using a commercially available radioimmunoassay kit with the Solid Phase Antigen Linked Technique (SPALT) with the LIAISON-Analyzer (DiaSorin, S.p.A., Italy). Plasma ACTH was analysed using an immunoradiometric assay (Immulite, 2500 ACTH, Germany). All hormone analyses took place in the endocrinology laboratory of the University Hospital of the Technische Universität Dresden. Due to the relatively short but intense treatment of 5 weeks with almost daily confrontation sessions, the DEX–CRH test was only performed before the start of the CBT.

### Statistics

Group comparisons with respect to sociodemographic and pre-therapy clinical variables were evaluated using univariate analyses of variance (ANOVA) for continuous variables and *χ*^2^-test for dichotomous variables. ANOVA for repeated measures were conducted to test for differences over time in the continuous clinical outcome measures of the PAS, ACQ, BSQ, and MI. Blood ACTH and cortisol data were subject to log transformation to reduce skewness. For ACTH analysis, data from one PD patient and from one healthy volunteer were excluded due to outlying values of more than three standard deviations above the mean. Group differences in the hormonal stress response (both for cortisol and ACTH) were assessed using a 2 (group: PD, healthy volunteers)×8 (time: −75, −1, 0, 10, 20, 30, 45, and 60 min) ANCOVA for repeated measures with baseline hormonal values (−75 min, −1 min) as covariates. To account for comorbid depression in 35.3% of the PD patients (*n* = 12), the BDI sum score was added as additional covariate. Greenhouse-Geisser corrections were used when necessary. Psychotherapy response was defined as at least 50% symptom reduction from pre- to post-therapy in the described questionnaires (except for the BDI). The DEX–CRH test *non-suppression* was defined as an increase of cortisol levels of more than 138 nmol/l compared to baseline^57^. Pearson’s correlational analyses (Bonferroni-corrected with *α* = 5%/7 tests = 0.7%) were conducted to quantify the associations between mean cortisol concentrations (of the eight time points of blood sample collection) and the psychotherapy outcome. For this, psychotherapy outcome scores were defined as percentage scores of the respective pre-therapy questionnaire scores, i.e., negative percentage scores indicate symptom reduction from pre- to post-therapy and positive percentage scores indicate an increase in symptomatology, respectively. All statistical analyses were performed using SPSS for Windows, version 22 (IBM, Chicago, IL). Data in figures are presented in original units and were plotted using Sigma Plot 11.0 for Windows (Systat Software, Inc., Erkrath, Germany).

## Results

### Sociodemographic and clinical measures

A brief description of the sociodemographic and pre-therapy clinical characteristics of the included study participants is provided in Table 1. Groups were well matched with regard to sociodemographic variables (*p*-values ≥ 0.156). As expected, patients diagnosed with PD with or without agoraphobia showed significantly higher scores in all panic-related questionnaires (*p*-values ≤ 0.052) and in depressiveness measured with the BDI (*p* ≤ .001). All the comorbid major depression diagnoses were present in those patients diagnosed with agoraphobia with PD who showed descriptively higher levels in depressiveness (mean ± SE: 13.14 ± 9.53) than those PD patients without agoraphobia (10.00 ± 4.58; *F*_1;31_ = 0.310; *p* = 0.582). According to the PAS self-report, 17.6% (*n = *6) of the patients showed borderline panic and agoraphobic symptoms, 23.5% (*n = *8) a mild, 41.2% (*n = *14) a moderate, and 17.6% (*n = *6) a serious disease severity. The mean duration of disease was 4.20 ± 5.10 years (range: 0–19 years).

**Table 1:** Characteristics of the total sample. Mean (SD) are listed except where noted

	Panic disorder patients	Healthy control subjects	*F/χ* ^2^	*p*-value
*Sociodemographics*				
Age (years)	35.50 (12.74)	33.82 (12.50)	0.300	0.586
Females, *n* (%)^†^	23 (67.6)	25 (73.5)	0.283	0.791
Body mass index	23.41 (3.66)	23.62 (2.51)	0.075	0.785
Smoking, *n* (%)^†^	16 (47.1)	13 (38.2)	0.541	0.624
Oral contraceptive use, *n* (%)^†^	8 (34.8)	10 (40.0)	0.065	1.000
Regular sport engagement, *n* (%)^†^	16 (47.1)	22 (64.7)	2.147	0.222
Sport engagement (h/week)	3.13 (2.13)	4.62 (3.53)	2.100	0.156
*Clinical measures*				
PAS total score [0–52]	19.53 (10.55)	1.06 (3.12)	95.783	0.000^***^
ACQ loss of control [0–4]	1.05 (0.67)	0.72 (0.73)	3.907	0.052^***^
ACQ physical concerns [0–4]	1.45 (0.66)	0.22 (0.54)	70.383	0.000^***^
ACQ total score [0–4]	1.25 (0.54)	0.47 (0.60)	32.042	0.000^***^
BSQ total score [0–4]	1.92 (0.73)	0.89 (0.59)	41.789	0.000^***^
MI alone [0–4]	1.10 (0.86)	0.17 (0.29)	35.475	0.000^***^
MI accompanied [0–4]	1.50 (1.09)	0.10 (0.22)	53.295	0.000^***^
BDI [0–63]	12.84 (9.19)	3.35 (3.05)	32.501	0.000^***^
*Therapy responder* ^1^				
PAS (%)	10 (29.4)	—	—	—
ACQ total score (%)	18 (52.9)	—	—	—
BSQ total score (%)	10 (29.4)	—	—	—
MI alone (%)	21 (61.8)	—	—	—
MI accompanied (%)	17 (50.0)	—	—	—

*PAS* Panic and Agoraphobia Scale, *ACQ* Agoraphobic Cognitions Questionnaire, *BSQ* Body Sensations Questionnaire, *MI* Mobility Inventory, *BDI* Beck Depression Inventory, *PASA* Primary Appraisal Secondary Appraisal Scale, *VAS* Visual Analogue Scale.^†^*χ*^2^-test. ^1^Response to psychotherapy was defined as at least 50% symptom reduction from pre- to post-therapy in the named questionnaire.***p ≤ 0.001

### Hormonal stress response to the DEX–CRH test

**Fig. 1:**
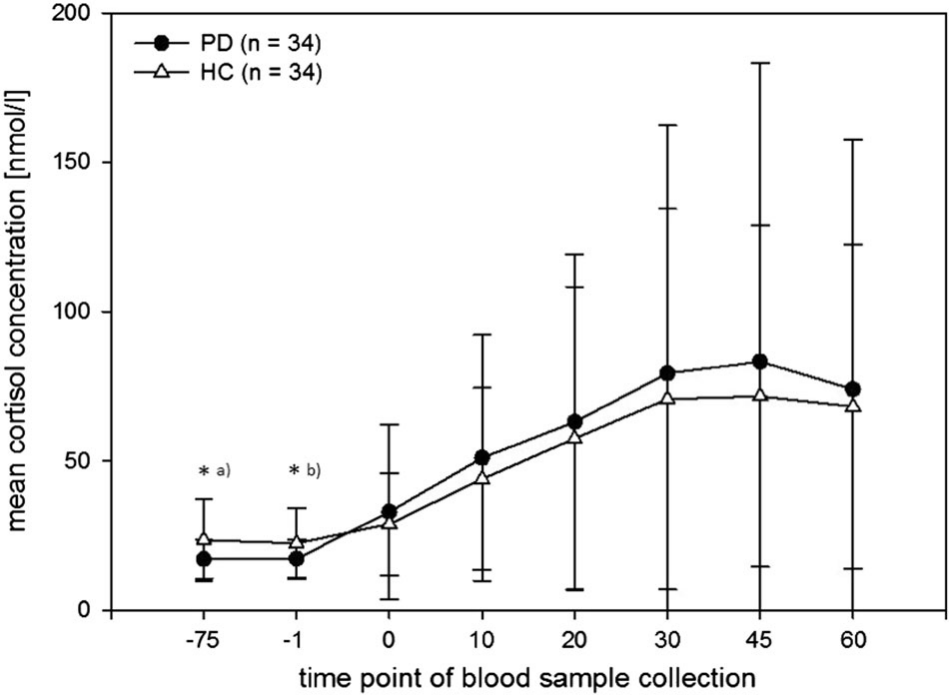
Mean (±SD) blood plasma cortisol concentrations in response to the DEX–CRH test in panic disorder patients with and without agoraphobia (PD) and healthy volunteers (HC) ^*^p ≤ .05. **a** mean ± SE, PD: 2.76 ± 0.41, HC: 3.04 ± 0.50; *p* = 0.016. **b** PD: 2.78 ± 0.38, HC: 3.00 ± 0.48; *p* = 0.040

There were no significant differences in the DEX–CRH suppression rates between the study groups (*χ*² = 0.108, df = 1, *p* = 1.000). 82.4% of the patients (*n* = 28) and 85.3% of the healthy volunteers (*n* = 29) showed a DEX–CRH suppression. Fig 1 illustrates the hormonal stress response to the DEX–CRH test as a function of study group. Univariate ANOVA revealed significant group differences in baseline cortisol levels measured 75 min prior to CRH-injection (patients: mean ± SE: 2.76 ± 0.41; healthy volunteers: 3.04 ± 0.50; *p* = 0.016) as well as in baseline cortisol levels measured 1 min prior to CRH-injection (patients: 2.78 ± 0.38; healthy volunteers: 3.00 ± 0.48; *p* = 0.040). No significant group differences in baseline ACTH levels both 75 min and 1 min prior to the CRH-injection were seen.

Repeated measures analysis of covariance (ANCOVA), with baseline cortisol levels and the BDI sum score added as additional covariates, revealed a significant main effect of time (*F*_1.815;108.889_ = 8.132, *p* ≤ 0.001, *η*^*2*^* = *0.119). Neither a significant time x group interaction effect (*F*_1.815;108.889_ = 0.823, *p* = 0.432, *η*^*2*^* = *0.014) nor a main effect of group (*F*_1;60_ = 0.089, *p* = 0.767, *η*^2^ = 0.001) could be observed. With respect to ACTH concentrations, repeated measures ANCOVA, with baseline ACTH concentrations and the BDI sum score added as covariates, revealed a significant main effect of time (*F*_1.990;117.416_ = 27.932, *p* ≤ 0.001, *η*^*2*^* = *0.321), while no time x group interaction effect (*F*_1.990;117.416_ = 0.516, *p* = 0.597, *η*^2^ = 0.009) nor main effect of group (*F*_1;59_ = 0.059, *p* = 0.809, *η*^*2*^* = *0.001) were seen.

### Psychotherapy outcome

ANOVA revealed highly significant reductions in all the panic-related self-report measures from pre-therapy to post-therapy with large effect sizes (*η*^2^ ≥ 0.14) ranging 0.321 ≥ *η*^2^ ≤ 0.547 (see Table 2 for details). Different therapy responder rates were seen ranging from 29.4% (measured with the PAS and BSQ) to 61.8% (measured with the MI) depending on the specific questionnaire used (see Table 1 for details). With regard to the PAS self-report after psychotherapy, 35.3% (*n = *12) of the patients showed borderline panic and agoraphobic symptoms, 26.5% (*n = *9) a mild, 29.4% (*n = *10) a moderate, and 5.9% (*n = *2) a serious disease severity.

**Table 2:** Clinical measures before and after psychotherapy for panic disorder patients

Clinical measure	Pre-therapy		Post-therapy		Statistics		
	Mean	(SD)	Mean	(SD)	*F*	*p*-value	*η* ^*2*^
PAS total score	19.33	(10.65)	14.01	(9.59)	15.161	0.000^***^	0.321
ACQ loss of control	1.05	(0.68)	0.57	(0.49)	20.145	0.000^***^	0.386
ACQ physical concerns	1.46	(0.66)	0.79	(0.65)	30.095	0.000^***^	0.485
ACQ total score	1.26	(0.54)	0.68	(0.52)	36.791	0.000^***^	0.535
BSQ total score	1.92	(0.73)	1.25	(0.71)	28.110	0.000^***^	0.468
MI alone	1.12	(0.88)	0.51	(0.59)	36.278	0.000^***^	0.547
MI accompanied	1.60	(1.10)	0.88	(0.87)	14.545	0.001^***^	0.334

*PAS* Panic and Agoraphobia Scale, *ACQ* Agoraphobic Cognitions Questionnaire, *BSQ* Body Sensations Questionnaire, *MI* mobility inventory.***p ≤ 0.001

**Fig. 2:**
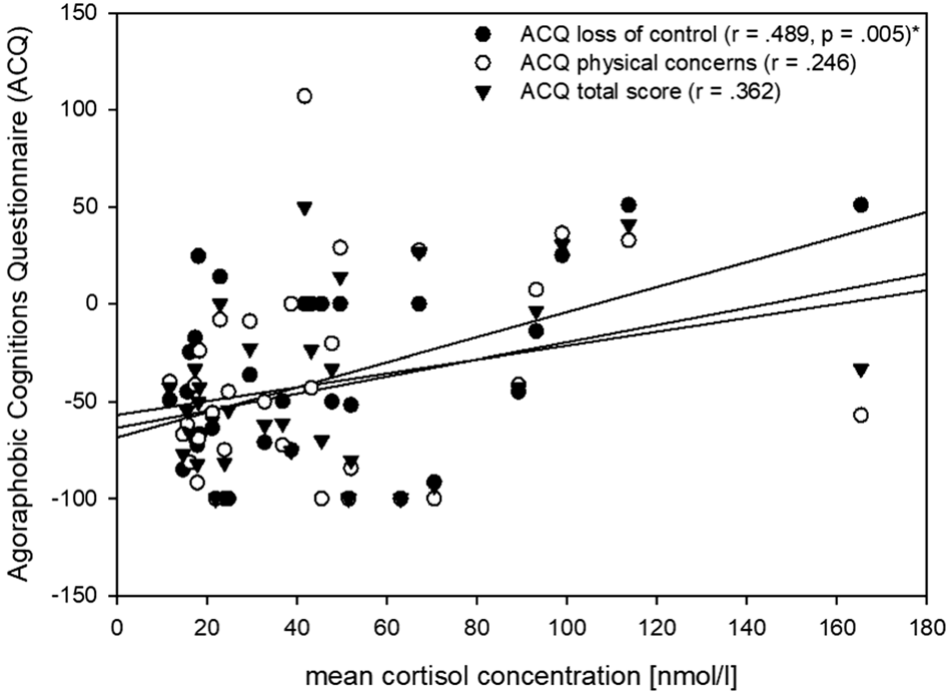
Scatterplot illustrating the relation between mean cortisol concentration in response to the DEX–CRH test and psychotherapy outcome percentage scores in agoraphobic cognitions for panic disorder patients; negative percentage scores illustrate symptom reduction *p ≤ 0.007 (Bonferroni-corrected)

The Pearson’s correlational analysis showed significant associations between mean cortisol concentrations and the ACQ loss of control outcome score (*r* = 0.489, *p* = 0.005; see Fig. 2). No significant associations were seen between mean cortisol concentrations and the PAS, ACQ physical concerns and total score, BSQ, and MI outcome scores (*p* ≥ 0.028).

## Discussion

We performed an intervention study of 5-week CBT in patients diagnosed either with agoraphobia with PD or PD without agoraphobia and exposure to the DEX–CRH test in both the patient and healthy control group. We predicted cortisol non-suppression in the patient group on the basis of our literature review. Concerning psychotherapy outcome, we analysed whether we would be able to replicate the inverse associations between the cortisol stress response and post-therapy disease severity which we had previously observed in a sample of PD patients confronted with the TSST.

CBT showed large effect sizes with significant reductions in all cardinal symptom clusters of PD (panic beliefs and agoraphobic cognitions, fear of bodily sensations, agoraphobic avoidance behaviour). Concurrently, the number of patients who reported a moderate or serious disease severity decreased (from 41.2 to 29.4% observed for moderate and from 17.6 to 5.9% for severe severity of illness), while the number of patients reporting borderline or mild severity increased, respectively (from 17.6 to 35.3% observed for borderline and from 23.5 to 26.5% for mild severity). Regarding psychotherapy success, those patients with *higher* cortisol concentrations upon CRH-injection showed percental increases in disease severity after CBT, therefore the least improvement by psychotherapy. We thus confirm former findings of an inverse correlation between cortisol concentrations and therapy success^12,13^. It seems that those patients who show inadequate hormonal responses show less improvement from psychotherapy. With regard to the TSST, an adequate and “normative” response pattern refers to a cortisol release of at least 2- to 4-fold elevations above baseline levels, as has been reliably reported for healthy individuals^58,59^. Patients of our previous trial who did not show a TSST stress response, thus displaying low cortisol concentrations upon psychosocial provocation, show concurrently a higher severity of disease and less improvement after therapy^13^. Siegmund et al.^12^ exposed the patients to situations feared individually and observed that patients who showed less cortisol concentrations during exposure had a worse therapy outcome. Results suggest that patients diagnosed with PD with or without agoraphobia fail to activate cortisol release when necessary. Adequate responding to the DEX–CRH test is indexed via cortisol suppression^15^. In the present sample, and that of Coryell et al.^30^, patients who showed adequate cortisol suppression showed concurrently higher therapy success than indexed in lower levels of disease severity. Specifically, in the present sample, the inverse correlation between cortisol levels and therapy outcome was preeminent in the loss of control scale of agoraphobic cognitions. Losing control/going crazy is a prominent feature in PD and results suggest that its activation during the therapy process holds a key role in psychotherapy success. This implication combines well with established recommendations for CBT for PD patients recommending a high level of fear^49^. The DEX–CRH test is an interoceptive hormonal stressor that PD patients might have experienced as an “interoceptive challenge” triggering panic-specific fears of a physical catastrophe and of losing control. PD patients usually show a heightened level of fear of interoceptive sensations resembling panic^60^ and report fear of medication intake due to possible side effects, for instance. The DEX–CRH test requires the oral intake of dexamethasone as well as CRH-injections. One might suggest that anticipatory fear of uncomfortable bodily sensations may be responsible for psychophysiological arousal and thus heightened baseline cortisol levels. However, this study didn’t include an anxiety rating to evaluate any discomfort in facing the DEX–CRH test, and we actually observed substantial lower baseline cortisol levels in PD patients. Furthermore, contrary to our expectations, most of the patients with and without agoraphobia did not show the expected non-suppression pattern, instead showing adequate cortisol stress responses comparable to the stress response pattern seen in the healthy volunteers. Though we included various stress-related variables in the sample characterisation, the differences mentioned in baseline cortisol levels may have resulted from a variable that we not assessed, e.g., overprotective parental behaviour^61^. Most of the study participants (>80% in both groups) demonstrated DEX–CRH suppression. This finding of DEX–CRH suppression concurs with previous studies who also failed to show an impact of the PD diagnosis on DEX–CRH suppression rates^36–38^. However, it conflicts, first, with reports of DEX–CRH non-suppression in PD patients^28–33,62^ and, secondly, with reports of substantial lower post-DEX–CRH cortisol levels in PD patients as compared to healthy volunteers^22,34^. Lower basal cortisol levels might have resulted from the habituation of cortisol releases from a chronically activated HPA axis. Some authors suggested that the alterations in the HPA axis that have been observed in some studies in contrast to the consistent hypo-responsive pattern might have resulted from anticipatory fear, not from spontaneous occurring panic attacks^32,63^. This mechanism could explain the relatively consistent pattern of a cortisol non-responsiveness upon provocation in PD patients^12,13,25,64,65^.

Previous psychotherapy research has primarily put an emphasis on psychological or disorder-related variables to predict the therapy success in PD patients (e.g., refs. 8,9,) resulting in no consistent associations. Implications of these study results may tentatively include the prediction of the therapy success from a potential endocrinological correlate whose persistency of inadequate cortisol suppression during treatment may predict response or non-response to the current treatment. However, this suggestion remains rather speculative. Future studies could include a second DEX–CRH test as well as a further rating of the disease severity to test if an improvement in HPA axis functioning (adequate cortisol suppression) was associated with a better treatment response. Further, if the improvement in HPA axis functioning precedes clinical improvement, this correlate may be used in addition to clinical and self-evaluation of the disease severity as a decision support. Such a study design could test the hypothesis if patients who don’t show improvement in the DEX–CRH-test were not likely to respond to the current treatment and probably require a change of the treatment strategy to avoid leaving patients under an inefficient treatment.

The limitations of this study refer to characteristics in the patient sample. First, our findings with respect to the association of the cortisol stress response and psychotherapy outcome may not extend to patients with a “pure” PD diagnosis or patients with other comorbid mental disorders. Second, this study was conducted with a relatively small sample size making it harder to detect significant differences. Finally, we did not include a follow-up assessment to explore whether the cortisol stress response is also predictive of long-term psychotherapy response. In conclusion, despite the named limitations, PD patients with and without agoraphobia demonstrated “normative” cortisol stress responses to the DEX–CRH test compared to healthy volunteers. An association between the cortisol stress response and psychotherapy outcome could be shown significantly for agoraphobic cognitions. Specifically, those patients who showed inadequate cortisol suppression demonstrated less improvement after CBT, especially concerning the fear of losing control. Implications for future studies are the recruitment of larger sample sizes and the implementation of an anxiety rating and a follow-up assessment.
